# HIV-1 Infection of DC: Evidence for the Acquisition of Virus Particles from Infected T Cells by Antigen Uptake Mechanism

**DOI:** 10.1371/journal.pone.0007470

**Published:** 2009-10-15

**Authors:** Narasimhan J. Venkatachari, Sean Alber, Simon C. Watkins, Velpandi Ayyavoo

**Affiliations:** 1 Department of Infectious Diseases and Microbiology, Graduate School of Public Health, University of Pittsburgh, Pittsburgh, Pennsylvania, United States of America; 2 Department of Cell Biology and Physiology, School of Medicine, University of Pittsburgh, Pittsburgh, Pennsylvania, United States of America; Beijing Institute of Infectious Diseases, China

## Abstract

Dendritic cells (DC) play a pivotal role in transmission and dissemination of HIV-1. Earlier studies reported that DC present at the site of infection trap virus particles via DC-SIGN and transfer the virus to the interacting naïve T cells. This prompted us to ask the question whether DC could acquire virus from infected T cells during DC-T cell interaction. To address this, we investigated the likely transfer of virus from HIV-1 infected T cells to DC and the underlying mechanisms involved. Results indicate that DC acquire virus from infected T cells via antigen uptake mechanism and this results in infection of DC with expression of proteins directed by viral DNA. Further studies with HIV-1 lacking the Env protein also resulted in infection of DC. The use of antibodies against DC-SIGN and DC-SIGN-R ruled out a role for receptor in the infection of DC. Additional data show that DC infection is directly correlated with the ability of DC to take up antigen from infected T cells. Overall, these studies provide evidence to suggest that HIV-1, besides infecting immune cells, also utilizes immunological mechanism(s) to acquire and disseminate virus.

## Introduction

HIV-1 infects macrophages, dendritic cells and T cells, which are also the key cells involved in inducing immune activation against invading pathogens [1], [2], [3]. HIV-1 transmission, infection and dissemination are facilitated by both cell-free and cell-associated virus *in vitro* and *in vivo* [4]. However, cell-associated virus transmission is more efficient than cell-free virus transmission [5], [6]. Thus, HIV-1 has devised several strategies to utilize this pathway. One of the ways HIV-1 enhances viral transmission is by converting the immunological synapse to a virological synapse between the interacting antigen presenting cell (APC), T cell, and other immune cells. Dendritic cells (DC) are one of the first targets that encounter virus at the mucosal surface during transmission *in vivo* [7], [8]. DC present under the mucosal membrane capture cell free virus as well as interact with infected donor cells, through the breached epithelial layer [1]. During this process DC capture virus particles and *trans* infect T cells efficiently as “Trojan Horses” [9]. In addition to the ability of DC to acquire virus in *trans*, a small percentage of DC are also infected in *cis* and support virus replication both *in vivo* and *in vitro* [10], [11], [12], [13], [14], [15]. Thus, DC play a key role in infection, virus dissemination and pathogenesis.

DC interact with pathogen infected/exposed cells in various tissue compartments as part of the immune surveillance function *in vivo* [16], [17]. DC uptake antigens (from both cell membrane and cytoplasm) from infected cells, process and present them to naïve and memory T cells. These studies indicate that there is sufficient interaction between DC and T cells during pathogen encounter. In HIV-1 infected individuals, activated CD4+ T lymphocytes are the major target cells for virus replication and infected T cells are present both in the periphery and in lymphoid organs [18], [19]. Previous studies report that when an infected DC interacts with an uninfected T cell, captured virus in DC is transmitted to the T cells, that results in productive infection of T cells [10], [20], [21], [22]. However, it is not known whether DC could acquire virus from an infected T cell resulting in infection of DC. To address this, we cocultured infected T cells with naïve DC and evaluated the infection of DC by HIV-1. For this purpose, we used a HIV-1 reporter proviral plasmid that codes for EGFP before the *nef* open reading frame as described [23]. The reporter virus derived from the plasmid has allowed us to measure the expression and subcellular distribution of EGFP (driven by HIV-1 LTR) only in infected DC.

Results presented here indicate that the cell-associated virus was taken by DC and infected DC as early as 12 hours and was maintained for more than six days, whereas cell free virus required 2–3 days to establish productive infection in DC. Infection of DC via infected T cell is dependent on T cell-DC contact and is independent of viral envelope and DC-SIGN. Furthermore, the percentage of DC infection is directly correlated with the ability of DC to acquire cell-associated antigen, suggesting DC could acquire virus from the infected T cells through the antigen uptake process. Collectively, these studies for the first time indicate that HIV-1 taken up by the DC through the antigen uptake mechanisms establishes *cis* infection in DC.

## Results

### Infection of DC mediated by cell associated virus

**Figure 1:**
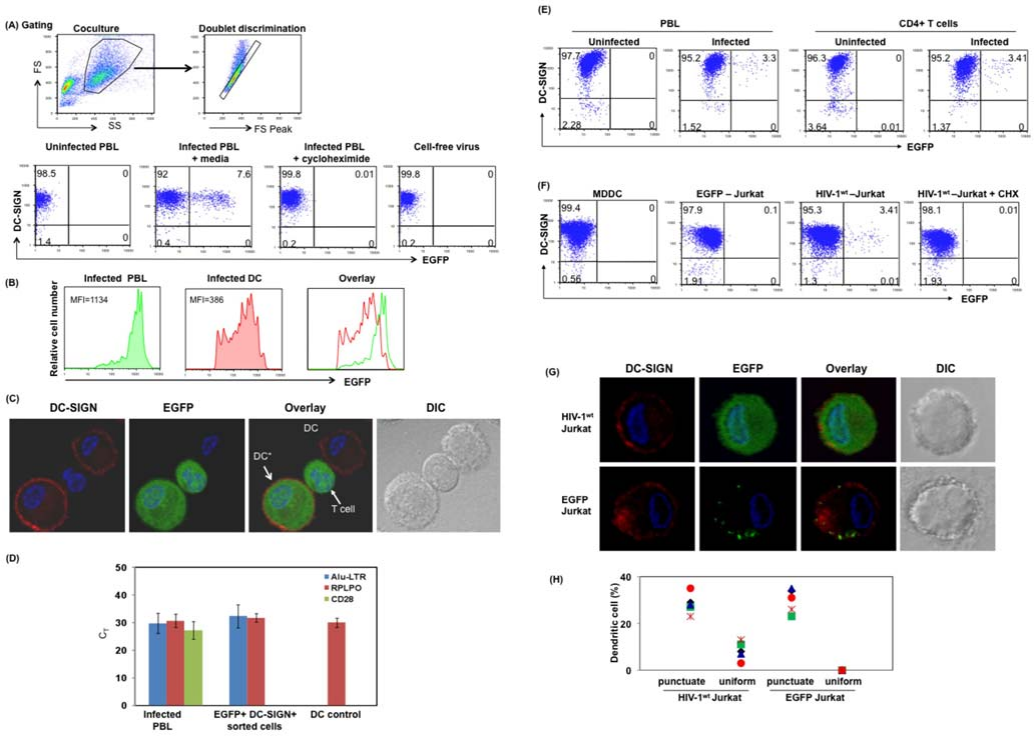
Reporter virus positive DC are the result of *cis* infection of DC. (A) DC were cocultured with HIV-1^wt^-EGFP reporter virus-infected PBL cells in the presence or absence of cycloheximide (10 µg/ml); or infected with cell-free virus. Post coculture (12 hrs), cells were stained for DC-SIGN. DC were gated based on side scatter and forward scatter followed by doublet discrimination (as shown in gating) and assessed for EGFP by flow cytometry. DC-SIGN and EGFP positive cells (%) are shown in the upper right quadrant. (B) Comparison of EGFP fluorescence (MFI) in infected lymphocytes and infected DC. Overlay of histogram of EGFP fluorescence in infected lymphocytes (green) and infected DC (red). (C) Detection of DC expressing EGFP by immunofluorescence microscopy. Red indicates DC-SIGN positive cells; green represents EGFP positive cells; Blue represents nuclear staining by DAPI. DC*, represents DC-SIGN and EGFP positive DC. (D) Detection of integrated HIV-1 proviral DNA in EGFP+ DC. DC were stained for DC-SIGN, and DC-SIGN+/EGFP+ DC were sorted by FACS. Integrated proviral DNA was assessed by real time Alu-LTR Taqman assay as described in Methods. To rule out contaminating lymphocytes in DC-SIGN+/EGFP+ sorted DC, mRNA from the sorted cells were evaluated for presence of CD28 mRNA by real-time PCR. RPLPO was used as control. Uninfected DC, Infected PBL controls were included. (E) DC were cocultured with HIV-1^wt^-EGFP reporter virus-infected PBL cells or with HIV-1^wt^-EGFP reporter virus-infected purified CD4+ T cells, or uninfected control cells. Twelve hours post coculture, cells were stained for DC-SIGN and assessed for EGFP positivity by flow cytometry. Cells (%) that are positive for DC-SIGN and EGFP are shown in the upper right quadrant. (F) DC were cocultured with either HIV-1^wt^-EGFP reporter virus-infected Jurkat T cells or with Jurkat cells expressing EGFP protein. Post coculture, the cells were stained for DC-SIGN and analyzed by flow cytometry or by (G) Immunofluorescence microscopy. DC-SIGN+/EGFP+ cells were gated based on the amount of EGFP in DC-SIGN+ cells to differentiate antigen uptake and productively infected DC. Results from multiple donors are shown in Fig. 1H, where 200 DC were counted scanning multiple fields (Fig. S2) for each culture. Figure represents one of 5–7 independent experiments with similar results.

DC generated as described in methods were cocultured with infected lymphocytes at a ratio of 2∶9∶1 (DC: uninfected PBL: infected PBL). Post coculture cells were stained for DC-SIGN, and EGFP+/DC-SIGN+ cells were determined by flow cytometry. DC were gated based on side scatter and forward scatter followed by doublet discrimination gating (Fig. 1A). Single cells that are double positive for DC-SIGN+ and EGFP+ were considered as productively infected DC (Fig. 1A). Results from coculture experiment indicate that 7.6% of DC were infected at 12 hours post coculture with infected lymphocytes, whereas cell free virus did not infect DC (0%) at this time point (Fig. 1A). Addition of cycloheximide (CHX) (10 µg/ml) during coculture completely blocked infection of DC further confirming that EGFP expression in infected DC was due to *de novo* synthesis, and not due to cell conjugates or cell fusion. Comparison of Mean Fluorescence Intensity (MFI) of EGFP in infected DC and infected lymphocytes present in the same coculture (Fig. 1B), indicates that transcription of HIV-1 LTR driven *EGFP* in infected DC is significantly less compared to infected lymphocytes. DC infection was further confirmed by fluorescence microscopy where, DC-SIGN positive cells were EGFP also positive (Fig. 1C) as identified by the uniform subcellular distribution of EGFP that is indicative of *de novo* synthesized EGFP. To further validate infection in DC, integrated proviral DNA was measured in EGFP+ DC. To assess integrated proviral DNA, DC-SIGN+/EGFP+ DC were sorted and assessed for integrated proviral DNA by real time Alu-LTR Taqman assay, and for CD28 mRNA by real-time PCR. Uninfected DC and infected lymphocytes were used as negative and positive controls, respectively. Results indicate that integrated DNA was detected by Alu-LTR Taqman assay in DC-SIGN+/EGFP+ DC (Fig. 1D). Additionally these cells were also negative for CD28 mRNA (Fig. 1D), further confirming that integrated proviral DNA detection in sorted DC was not due to contamination of infected T cells in the culture. Together these results indicate that EGFP expression in DC is due to integrated proviral DNA that is indicative of *cis* infection. Similarly purified CD4+ T lymphocytes infected with the HIV-1^wt^-EGFP reporter virus also infected DC in cell-associated manner (Fig. 1E).

DC are known to take up antigens/apoptotic cells by endocytosis, micropinocytosis and other mechanisms [24], [25]. Therefore, we next delineated the uptake of cellular materials, including EGFP protein from the infected T cell versus *de novo* synthesis of EGFP in DC. DC were cocultured with either HIV-1^wt^-EGFP reporter virus-infected Jurkat T cells or with Jurkat cells expressing EGFP protein by transient transfection and assessed by flow cytometry. Results presented in Fig. 1F indicate that 3.41% DC are positive for EGFP following 12 hours of cocultured with HIV-1 infected cells. We also confirmed EGFP synthesis versus EGFP uptake in DC by fluorescence microscopy (Fig. 1G and Fig. S2) and observed a uniform cytoplasmic and nuclear distribution of EGFP in infected DC (upper panel), whereas, punctuate pattern was noted in DC following EGFP uptake (bottom panel). Similar results were observed in multiple donors (Fig. 1H). The difference seen in the amount of EGFP in DC taking up the antigen and infected DC was not due to differences in the amount of EGFP in the cocultured Jurkat-EGFP cells or infected Jurkat cells (Fig. S1D). Together these results indicate that, EGFP+ DC seen in coculture experiment are due not to EGFP (antigen) uptake but rather it is due to *de novo* synthesis of EGFP in infected DC.

### Productive infection of immature and mature DC is cell contact dependent

**Figure 2:**
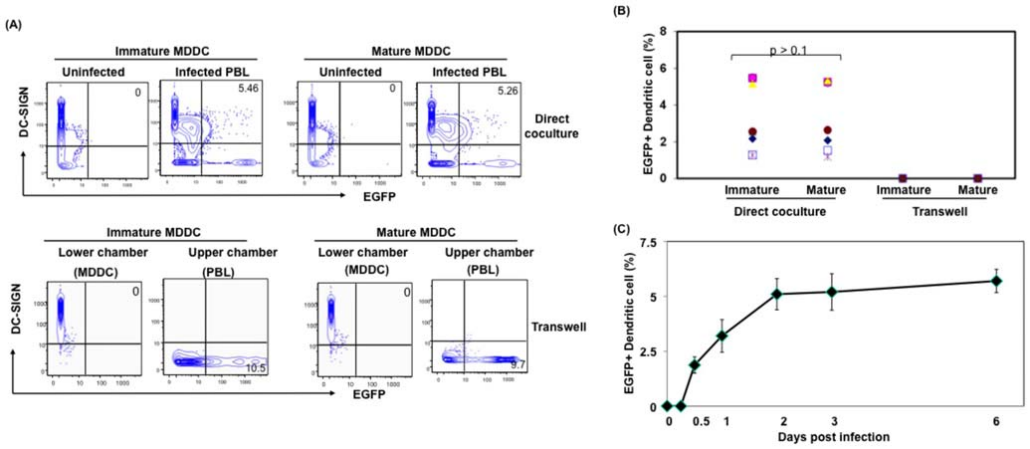
Infection of immature and mature DC by infected lymphocytes is cell-to-cell contact dependent. (A) Immature and mature MDDC were cocultured with HIV-1^wt^-EGFP virus infected PBL at a ratio of 2∶9∶1 (DC:uninfected PBL:infected PBL) either directly or separated by a transwell. Post coculture (12 hours) cells were stained for DC-SIGN, and analyzed by flow cytometry. Gating was extended to include lymphocytes and doublet differentiation was applied. (B) Cell contact dependent productive infection of immature and mature DC by lymphocyte associated HIV-1 virus in multiple donors (n = 8). (C) Time kinetics of productive infection in DC mediated by T cell associated virus. The figure is representative of data acquired from multiple donors (n = 5). Error bars indicate S.D. of the results obtained from triplicate wells from a single donor.

Next, to assess whether both immature and mature DC could be infected with cell-associated virus, immature and mature DC were cocultured with infected lymphocytes as described in Fig. 1. Additionally, to differentiate the role of cell free and cell-associated virus in DC infection, infected T cells were separated from DC via a transwell with a pore size of 0.4 µm which will allow free virus released from infected lymphocytes in the upper chamber to pass to DC in the lower chamber, but prevent contact between infected T cells and DC. Results indicate that no EGFP+ DC (0%) when they were separated by transwell, whereas, 5.2% EGFP+DC-SIGN+ DC was observed in mixed culture (Fig. 2A). Similar results were observed in multiple donors (Fig. 2B), suggesting that cell-to-cell contact is necessary for DC infection within 12 hours. Additionally, DC from the same donors infected with cell-free virus did not show productively infected DC at the same time point (data not shown). Time course analysis indicates that DC-SIGN+/EGFP+ cells remained positive for EGFP up to 6 days post coculture (Fig. 2C). Additionally, cell-free virus released from the infected T cells reaching the lower chamber established infection (<0.2%) only in immature DC 3 days post exposure (6–8 infected cells per 20 high power fields were detected by microscopy). Similar low level cell-free virus mediated DC infection was reported previously [20], [26]. These results clearly indicate that the accelerated infection of both mature and immature DC mediated by infected T cell is contact dependent and is not the consequence of cell free virus infection. Since the pore size of transwell is 0.4 µm, it further rules out the involvement of exosomes derived from infected T cells in infecting DC. It is important to note that, though we observed infected DC in multiple donors, there was a wide range in percentage of infected DC (2–8%), suggesting that the variation is due to difference in the susceptibility and/or permissibility of DC from different donors to support HIV-1 infection.

### Infection of DC mediated by cell-associated virus does not involve DC-SIGN, Mannose Receptor, CD4 or HIV-1 envelope

**Figure 3:**
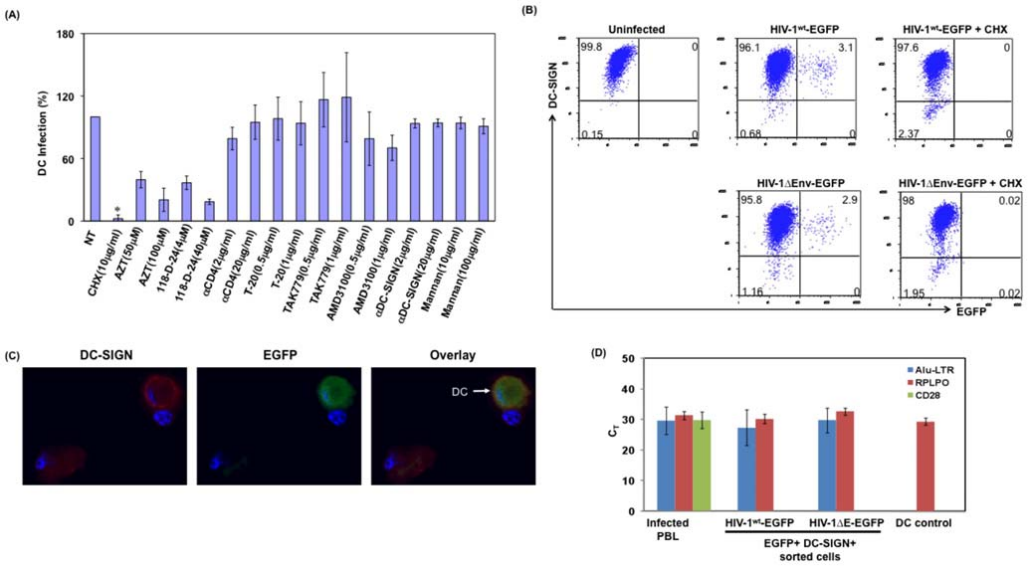
Infection of DC mediated by T cell associated virus is independent of viral envelope. (A) DC were cocultured with HIV-1^wt^-EGFP reporter virus-infected PBL cells in the presence of mentioned inhibitors. Post coculture (12 hours), cells were stained for DC-SIGN. Single DC were gated and assessed for EGFP by flow cytometry. For comparison across donors, the percentage of DC infection in absence of inhibitor was considered as 100%. Error bars indicate S.D of results obtained from results from multiple donors (n = 3). *, denotes p<0.5. (B) CD4+ T cells were infected with HIV-1^wt^-EGFP reporter virus or with HIV-1ΔE-EGFP reporter virus complemented with VSV-G Env expression plasmid. Three days post infection cells were washed thoroughly and cocultured with immature MDDC in the presence or absence of cycloheximide (10 µg/ml). Post coculture (12 hours), cells were stained for DC-SIGN and analyzed by flow cytometry, and (C) Immunofluorescence microscopy. Data are representative of five independent experiments. (D) Detection of integrated HIV-1 proviral DNA in EGFP+ DC by Real Time Alu-LTR Taqman assay, following twelve hours of coculture with CD4+ T cells infected with HIV-1ΔE-EGFP reporter virus complemented with VSV-G envelope. DC were stained for DC-SIGN, and DC-SIGN+/EGFP+ DC were sorted by FACS sorter. Integrated proviral DNA was assessed by real time Alu-LTR Taqman assay as described in Methods. To rule out contaminating lymphocytes in DC-SIGN+/EGFP+ sorted DC, mRNA from the sorted cells were evaluated for presence of CD28 mRNA by real-time PCR. RPLPO was used as control.

DC-SIGN and related C-type lectin receptors are suggested to play a role in *cis* and *trans* infection of DC, as blocking these receptors inhibits infection [20], [27], [28]. Therefore, we evaluated the ability of anti-DC-SIGN, anti-DC-SIGN-R antibodies, Mannan, anti-CD4 antibody, T-20 Fusion inhibitor, HIV-1 co-receptor antagonists TAK 779 and AMD 3100 to block DC infection mediated by cell-associated virus. AZT and Intergase inhibitor (118-D-24) were used as control to inhibit virus replication. Additionally cycloheximide (10 µg/ml) was used as a control for *de novo* synthesis of EGFP in infected DC. As shown in Fig. 3A, HIV-1 receptor and the co-receptor blockers failed to prevent infection of DC mediated by infected T cells, whereas AZT and Integrase inhibitors blocked infection by 67% and 83% at higher concentrations of 100 µM and 40 µm respectively, compared to untreated control. However they do not block the infection as seen in cell-free virus infection. Similar results were observed in multiple donors (n = 5). Together, these results suggest that DC-SIGN, DC-SIGN-NR, Mannan receptors, CD4 or the HIV-1 co-receptors are not involved in T cell mediated infection of DC.

To assess whether presence of HIV-1 envelope is required for virus transmission from infected T cell to DC, we used HIV-1ΔEnv-EGFP virus infected T cells (pseudotyped with VSV-G envelope expression plasmid to infect T cells). Three days post infection, cells were washed thoroughly and cocultured with DC as described above and assessed for DC-SIGN+/EGFP+ cells (Fig. 3B). Results indicate that DC were infected with HIV-1ΔEnv-EGFP virus as determined by flow cytometry in multiple donors (n = 4). Flow cytometry results were validated by fluorescence microscopy (Fig. 3C) and by real time Alu-LTR Taqman assay (Fig. 3D). Experiments to determine the presence of contaminating lymphocytes by evaluating the presence of CD28 mRNA in the sorted, infected DC indicate no detectable CD28 mRNA, further ruled out lymphocytes contamination. Collectively, these results support that cell-associated infection of DC is independent of both DC cell surface receptors and viral envelope.

### Infection of DC is directly correlated with the ability of DC to acquire cell-associated antigen from the interacting cell

**Figure 4:**
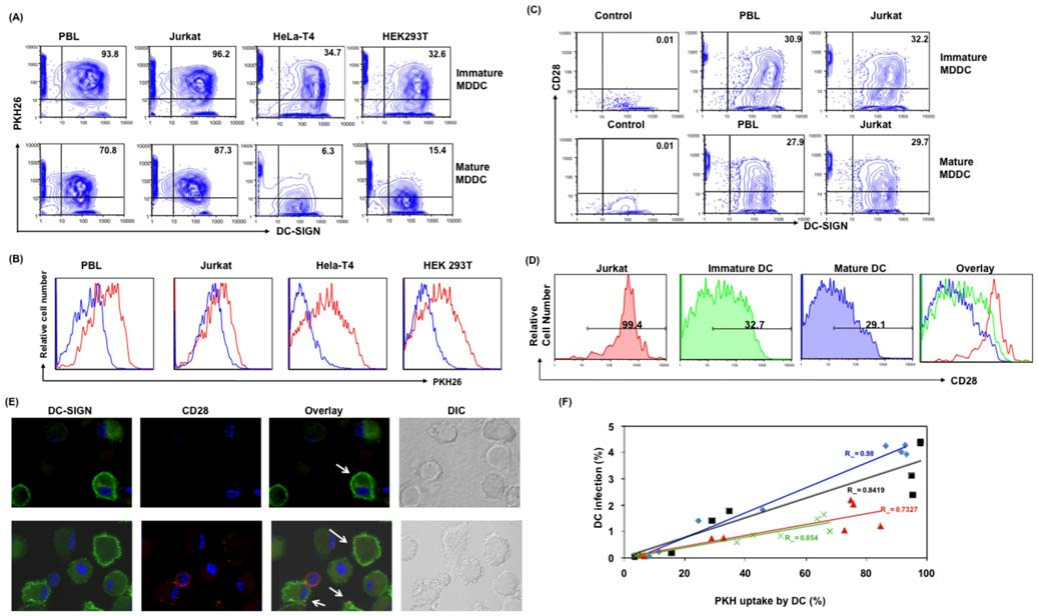
Infected T cell mediated DC infection is directly correlated with the ability of DC to acquire antigen from T cell. (A) Immature and mature MDDC were cocultured with PKH26-labeled PBL, Jurkat cells, HeLa-T4 or HEK 293T at a ratio of 1∶1. Post coculture (12 hours) cells were stained for DC-SIGN and the amount of PKH26 uptake by DC-SIGN positive cells were assessed by flow cytometry. DC were gated based on side scatter and forward scatter followed by doublet discrimination. Values in upper right quadrant indicate the percentage of DC-SIGN and PKH26 positive cells. (B) Histogram overlay represent PKH26 fluorescence in immature (Red) and mature (Blue) DC, post cocultured (12 hours) with PKH-26 labeled PBL, Jurkat cells, HeLa-T4 or HEK 293T cells. The figure is representative of data obtained from experiments in four separate donors. (C) Immature and mature DC cocultured (for 12 hours) with PBL or Jurkat T cells were stained for DC-SIGN and CD28 and evaluated by flow cytometry. (D) Comparison of MFI of CD28 molecule on the surface of Jurkat, immature DC or mature DC cocultured with Jurkat T cells. (E) Immature DC were cocultured with Jurkat T cells for twelve hours, cells were stained for DC-SIGN and CD28 and evaluated by Immunofluorescence microscopy, green represents DC-SIGN, CD28 is depicted in red and DAPI staining of nucleus is shown in blue. DC alone control was included. (F) Scatter plot denotes the correlation of PKH26 uptake and DC infection post coculture with different cell types. Each color in the plot denotes individual donor (n = 4). Linear regression was calculated for each donor, along with the R^2^ value.

Results presented above indicate that transfer of virus from T cells to DC is independent of viral envelope as well as cell surface receptors in DC, suggesting that receptor independent mechanisms may be involved in virus transfer. DC are known to acquire surface molecules and cell-associated antigens from interacting cells [29], [30]. To understand whether antigen uptake mechanism is involved in virus transfer from infected T cell to DC, we examined the interrelationship between antigen uptake and DC infection. Immature and mature DC were cocultured with uninfected PKH26-labeled or infected PBL, Jurkat cells, HeLa-T4 or HEK 293T at a ratio of 1∶1. DC were assessed for PKH26 uptake, and infection (EGFP+) by flow cytometry. As shown in Figure 4A, 93.8% of immature DC acquired PKH26 labeled material from PBL, 96.2% from Jurkat, 34.7% from Hela-T4 and 32.6% from HEK 293T cells. Whereas, mature DC cocultured with PKH26 stained cells efficiently acquired PKH26 labeled material from PBL (70.8%) and Jurkat cells (87.3%) but failed to acquire PKH26 labeled material efficiently from HeLa-T4 cells (6.3%) or HEK293T cells (15.4%). Results indicate that immature DC acquired membrane from tested cell types to different proportions. Although the percentage of immature and mature DC positive for PKH26 is similar in case of PBL and Jurkat, it is important to note that the amount of PKH26 acquired by mature DC was lower than immature DC as seen by the MFI (Fig. 4B). These results indicate that both immature and mature DC uptake antigen from T cell lineage more efficiently and equally, whereas they exhibit differential ability to uptake antigen from epithelial cells.

In addition to cytoplasmic and membrane bound antigen uptake, APC are known to acquire membrane from the interacting cell surface on to their own surface in the right orientation, a phenomenon described as trogocytosis [31], [32], [33]. To understand whether DC infection is mediated through this mechanism, we evaluated the ability of DC to acquire T cell surface molecules, CD3 and CD28, by flow cytometry. We performed surface staining of these molecules using CD3 or CD28 specific antibodies, which will specifically detect these molecules, if they orient on the outer side of the membrane via trogocytosis. Results indicate that both immature and mature DC acquired CD28 from the interacting PBL or Jurkat (Fig. 4C). This observation was further confirmed by confocal microscopy by staining DC with anti-CD28 antibody. Results indicate that the presence of CD28 on DC cell surface, where speckles of CD28 was identified (Fig. 4D). Together these results indicate that DC acquire membrane from their interacting T cells via antigen uptake and trogocytosis.

When we compared the infection of DC within these cultures, we also observed that there is a direct correlation between membrane uptake and infection. In multiple donors (n = 4), at 12 hours following coculture at a ratio of 1∶1 (DC: PBL) (10% of PBL are infected) it was observed that both immature and mature DC were infected at the highest in case of PBL (2.7±1.1) and Jurkat (2.6±1.8) coculture, whereas, it was almost half when immature DC were cocultured with HEK293T (1.3±0.54) and HeLa-T4 (1.1±0.35) cells. Interestingly, mature DC did not show any infection when cocultured with HEK293T (0.16±0.07) or HeLa-T4 (0.08±0.04) cells and is directly correlated with the low/no antigen uptake. Statistical evaluation between PKH26 uptake by immature and mature DC from different cell type (PBL, Jurkat, Hela-T4, HEK 293T) and associated DC infection in multiple donors (n = 4), indicates a direct correlation between antigen uptake and infection of DC, R^2^ value ranges from 0.98 to 0.73 (Fig. 4F). Together these results support that there is a strong correlation between antigen uptake and infection of DC via T cell-associated virus.

### Blocking the ability of DC to acquire cell-associated antigen prevents DC infection

**Figure 5:**
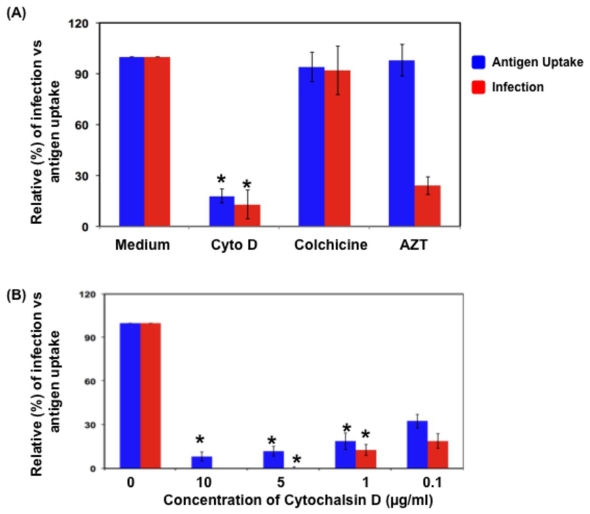
Blocking the ability of DC to acquire cell associated antigens prevents DC infection. (A) DC were cocultured with infected T cells in the presence of various inhibitors (Cytochalasin D 1 µg/ml; Colchicine 100 µg/ml; AZT 100 µM), post coculture (12 hours) were stained for DC-SIGN and the amount of antigen uptake by DC and percentage of productive infection of DC were evaluated by flow cytometry. The figure is representative of data obtained from one of the five independent donors. Error bars indicate S.D. of the results obtained from triplicate wells from a single donor. (B) Ability of Cytochalasin D to inhibit antigen uptake and productive infection of DC in dose dependent manner. The figure is representative of data obtained from one of four independent experiments. * denotes p<0.5.

As the ability of DC to acquire cell associated antigen and infection of DC is directly correlated, we further investigated whether blocking the membrane uptake will result in loss of DC infection. DC were cocultured with infected PBL in the presence and absence of cytochalasin D, Colchicine, AZT and evaluated for membrane uptake as well as DC infection (Fig. 5A). Results indicate that cytochalasin D blocked both membrane uptake and infection (>80%) significantly, whereas Colchicine did not show any effect of the membrane uptake or infection compared to the no treatment group. In case of AZT, it did not affect membrane uptake, whereas, it inhibited infection in DC by 75–80% at 100 µM compared to untreated group.

Furthermore, results using various concentrations of cyctochalasin D indicate that cytochalasin D inhibited membrane uptake as well as DC infection in a dose dependent manner (Fig. 5B). At a concentration of 0.1 µg/ml, the antigen uptake was reduced by 67.6±4.7% and the infection was inhibited by 71.1±5.1%, where as, at 1 µg/ml, the antigen uptake was reduced by 81.3±5.7% and the infection was inhibited by 87.3±3.8%. Cytochalasin D at concentrations of 5 and 10 µg/ml inhibited the antigen uptake to more than 90% and at these concentrations there was complete inhibition of infection in DC (Fig. 5B). Inhibition of infection was independent of cytotoxicity induced by cyctochalasin D as confirmed by annexin V staining in DC (data not shown). Together these results indicate that DC might acquire virus from infected T cells through the antigen/cell membrane uptake mechanisms. However, there may be other molecules/pathways such as immunological and virological synapses may be affected by cytochalasin D.

## Discussion

Both cell-free and cell-associated virus facilitates transmission, spread and dissemination of HIV-1. However, cell-associated virus infection has added advantages and is more effective than cell-free virus infection. Viral dissemination through cell-to cell contact is mediated through virological synapses, and this event is predominant at the secondary lymphoid organs [34], [35]. Several host cellular proteins, such as ICAM, LFA, ZAP-70 are known to regulate this event [36], [37], [38], [39], [40]. One of the ways these cellular proteins regulate virus transmission is through enhancing cell-to cell contact, ability these proteins to incorporate in the virus particles, suggesting that viruses utilize several modes for efficient transmission. Published studies also indicate that antiviral drugs, AZT or neutralizing antibodies do not block the cell-associated HIV transfer and infection completely, opposed to cell-free virus infection [41], [42]. Similarly, we observed reduction in inhibition at higher concentrations, whereas lower concentrations (that are required to block cell-free virus) did not show significant inhibition. Together these findings suggest that virus transmission also occurs through other mechanism(s) that are not well established.

In this study, we have shown for the first time that DC acquire virus from infected T cells utilizing the antigen uptake mechanisms that results in DC infection. Previous studies have focused on DC handing off virus to the interacting naïve T cells as part of the “Trojan horse” model that results in productive infection [20], [21]. However, it is not well understood whether a reverse phenomenon is possible. This is important as DC interact with infected T cells to sample foreign antigens for priming naïve T cells. Our results indicate that DC acquire virus from the infected cells during the antigen uptake process that results in DC infection. Although immature DC efficiently uptake antigen (>90% of total DC cocultured) from T cells, infection of DC is 5–8% of the total DC cocultured, suggesting that a small amount of virus is able to escape the antigen processing pathway and establish infection. The other possibility is that most of the budding virus particles that are transferred from infected T cell to DC may not be mature as part of the virion maturation occurs post release. A recent study by Turville et al [43], further support our finding that DC can take up virus from another infected DC. Envelope independent HIV-1 infection has been reported previously, in case of cells that lack CD4 receptors [44], [45]. Also the envelope deficient virus was able to transfer from infected DC to neighboring uninfected DC [43].

This phenomenon might have a significant impact *in vivo*, as DC and T cells interact at multiple sites including the site of entry (mucosal tissue) and lymphoid structures within the infected host. Upon infection by pathogens, various immune cells (infected and bystander) come in contact at the lymphoid tissues for antigen uptake, presentation, priming and induction of immune responses. Many of these processes occur through the formation of immunological synapses [46], [47], [48]. Pathogens, including HIV-1 are known to dysregulate immunological synapse and enhances virological synapse by differentially regulating viral and host cellular factors [7], [34], [49]. Utilizing this immunological process, DC capture free virus and efficiently *trans infect* T cells *in vivo* and *in vitro*. *In vivo* studies (animal model) indicate the presence of productively infected T cells 3 days post-intravaginal inoculation [50], [51]. Here we address the consequence of the interaction between an infected T cell and uninfected DC and its potential role in viral dissemination.

**Figure 6:**
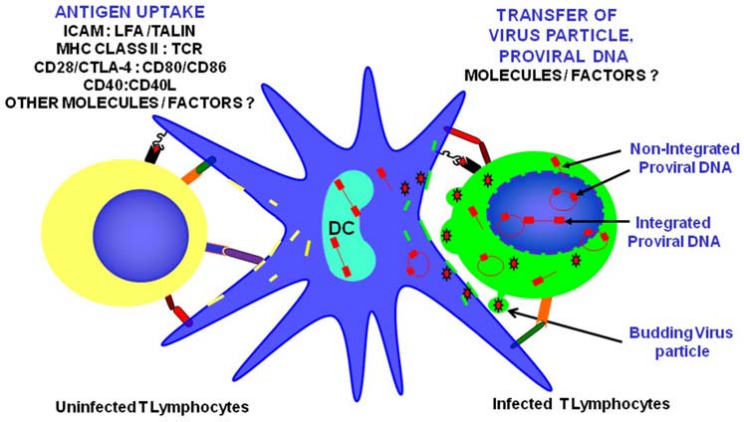
Proposed model depicting the various mechanisms(s) involved in virus transfer from infected T cell to DC. This includes the following potential mechanisms: Uptake of budding virus particle from the infected T cell via cell membrane uptake; Uptake of various forms of infectious unintegrated viral DNA from infected T cell cytoplasm via cytoplasmic antigen uptake; Membrane transfer of assembling and budding virus from the infected T cell and reorient on DC membrane; and Uptake of cellular and nuclear content including viral antigens and viral nucleic acids from the apoptotic infected T cells. DC is depicted in the middle; Cell with green cytoplasm denotes infected (EGFP+) T cells with various forms of unintegrated proviral DNA (shown in Red). Potential cell surface interacting receptors and ligands are marked in the T cells on the left.

Results presented here indicate that DC acquire virus from infected T cells independent of cellular and viral receptors that are involved in typical cell-free infections but dependent of cell-to-cell contact, suggesting that cell-to-cell communication and/or cellular networks are involved in virus transfer. Although results presented above indicate that DC might acquire virus from infected cells, via their antigen uptake mechanisms, it is not clear what kind of material transfer occurs between T cell and DC. Based on the cell surface ligands, their receptors involved in DC-T cell interaction, it is possible to predict that the presence of these costimulatory molecules might increase the affinity of cell-to cell contact though their ligands present in DC. Based on the available information, we proposed several scenarios that could be the source for DC infection as shown in Fig. 6. These include: (a) uptake of budding virus particle from the infected T cell via cell membrane uptake; (b) uptake of various forms of infectious unintegrated viral DNA from infected T cell cytoplasm via cytoplasmic antigen uptake; (c) membrane transfer of assembling and budding virus from the infected T cell and reorient on DC membrane; and (d) uptake of cellular and nuclear content including viral antigens and viral nucleic acids from the apoptotic infected T cells. Alternatively, virus could also transfer from cell to cell via a cellular network including nanotubules, and other related extensions, as DC acquire dyes, bacteria and other pathogens from cells through nanotubules [24], [52]. Transfer of viral nuclear material, either unintegrated viral DNA as LTR circles or linear proviral DNA may have an effect in viral pathogenesis. It should be noted that these unintegrated forms have varied half life, the linear unintegrated proviral does not survive for long, but conflicting reports suggest that the LTR ring forms may persist in the cells and may have a role in virus persistence, even in patients on HAART for a long duration. Alternatively DC can acquire immature virus particle, which may not be infectious. *In vivo*, DC scavenges the tissues for foreign antigens as part of normal immune surveillance. If HIV-1 utilizes these normal DC cell functions for virus transmission, this will have significant impact on pathogenesis and disease progression. Clearance of virus from the infected host will be much more difficult. Understanding the mechanism(s) involved in contact dependent virus transfer will further enhance our knowledge towards developing additional antiviral strategies to prevent HIV-1 transmission.

## Materials and Methods

### Cell Culture

Peripheral blood mononuclear cells (PBMC) were isolated from heparinized blood obtained from normal donors (through Central Blood Bank, Pittsburgh following approved protocols) using Ficoll-Hypaque gradient centrifugation. CD14+ monocytes were purified by positive selection using anti-CD14 monoclonal antibody-coated magnetic microbeads (Miltenyi Biotech, Auburn, CA) as described previously [53]. The purity of CD14+ cells was tested by flow cytometry following staining with CD14 antibody, and the results indicate >98% of isolated cells were CD14 positive (data not shown). To obtain monocyte-derived DC (MDDC), CD14+ cells (0.5×10^6^ cells/ml) were cultured in 60-mm culture plates in a total volume of 10 ml of medium containing 25 ng/ml IL-4 (R&D Systems, Minneapolis, MN) and 50 ng/ml granulocyte-macrophage colony-stimulating factor (GM-CSF) (R&D Systems). Half the volume of medium was replaced every other day throughout the entire culture period. MDDC (7 day old) were stimulated with LPS (Sigma-Aldrich) 1 µg/ml, and maturation of MDDC was confirmed by phenotypic and functional analysis (Fig. S1A, B). The flow through during the CD14+ selection of PBMC (same donor) was collected, and the purity of the lymphocytes (PBL) was tested by flow cytometry using CD14 and CD3 antibody. More than 95% of isolated cells were CD14− (data not shown). PBL (1×10^7^/ml) were stimulated with anti-CD3 (OKT3) antibody (10 µg/ml)-coated flasks along with soluble anti-CD28 antibody (BD Pharmingen clone 28.2) (1 µg/ml) for 3 days as described [54] and cultured in media containing rIL-2 (5 U/ml). CD4+ lymphocytes were isolated by negative selection from PBMC, more than 95% of isolated cells were CD4+ as tested by flow cytometry. Stimulation and infection of CD4+ T cells was performed in a similar way as PBL. Jurkat T cell line JJK, HeLa-T4 and HEK 293T cells were maintained in appropriate growth media.

### Plasmids, virus preparation and infection

The construction and characterization of HIV-1 wt-EGFP proviral plasmid has been previously described [23]. Briefly, Enhanced Green Fluorescence Protein (*EGFP*) gene was inserted in the *nef* open reading frame of pNL4.3 proviral plasmid and the expression of *nef* was driven by ECMV Internal Ribosomal Entry Site (IRES). For studies involving envelope deficient HIV-1ΔE-EGFP virus, NdeI site in the envelope of HIV-1^wt^-EGFP proviral plasmid was mutated by filling and re-ligating the blunt ends. This introduced multiple stop codons in the reading frame of env after the end of *vpu* reading frame, and deleted the expression of *env*. HEK293T cells (2×10^6^ per plate) were transfected with 10 µg of HIV-1wt-EGFP construct by calcium phosphate precipitation method [53]. Forty-eight hours post transfection supernatants were collected, filtered through a 0.45-µm filter to remove cellular debris, and centrifuged at 22,000 rpm for 1 h. Virus pellets were resuspended in PBS and stored in aliquots at −80°C for subsequent assays. Virus titers were measured by p24 enzyme-linked immunosorbent assay (ELISA), and multiplicity of infection (MOI) was calculated by infecting Jurkat cells for 24 hours and assessed by flow cytometry or by standard TZM-bl assay. PBL and Jurkat were infected with the HIV-1^wt^-EGFP reporter virus at a MOI of 0.5. Twelve hours post infection, virus was removed by washing, and cells were maintained in appropriate media. For infection of HEK293T and HeLa-T4 cells, the HIV-1^wt^-EGFP reporter virus was pseudotyped with VSV-G envelope and the cells were infected at a MOI of 1.0. Six hours post infection, the virus was removed by washing and the cells were maintained in growth media. VSV-G pseudotyped HIV-1ΔE-EGFP virus was produced by co-transfecting HEK293T cells with VSV-G and HIV-1ΔE-EGFP constructs by calcium phosphate precipitation method [53]. Forty-eight hours post transfection supernatants were collected, processed and the virus was quantitated as described above. PBL and Jurkat were infected with the HIV-1wt-EGFP reporter virus at a MOI of 0.5. Twelve hours post infection, virus was removed by washing, and cells were maintained in appropriate media. Transfection of HIV-1ΔE-EGFP construct in the absence of VSV-G produced non-infectious virus like particles. Similarly the virus particles released from the T cells in the supernatant were not infectious as evaluated by standard TZM-bl assay.

### Transfection of Jurkat

Jurkat T cell line JJK (CD4+/CD28+), were nucleofected with pEGFP, plasmid expressing EGFP using Amaxa nucleofector system, Amaxa Biosystems, Gaithersburg, MD following manufacturer's instructions. Briefly, the cells were washed and resuspended in RPMI medium without any supplements at a concentration of 5×10^6^, and 5 µg of plasmid was used to transfect the cells using appropriate settings. Following nucleofection, cells were maintained in RPMI supplemented with 10% FBS and 1% L-glutamate with no antibiotics.

### Flow cytometry

In coculture experiments, doublet differentiation was applied to gate on single cells. Surface staining was performed for DC-SIGN, in some of the experiments surface staining of CD3 or CD28 was also included. Briefly, at indicated time points cells were washed twice with cold PBS (pH 7.2) containing 5% FBS and incubated with respective fluorochrome conjugated antibody or isotype control for 1 h at 4°C. To minimize cell aggregates, 5 mM EDTA was included in the FACS buffer. Samples were fixed with 2% formaldehyde for 1 hr and analyzed using Epics-XL (Beckman Coulter, Miami, FL) with minimum of 20,000 gated events acquired for each sample. Flow Jo software was used to analyze the results.

### Fluorescence labeling

Cells were labeled with membrane labeling dye PKH26 (Sigma-Aldrich), according to the manufacturer's instructions. Briefly, the cells were washed twice in PBS and resuspended in Diluent C at a concentration of 2×10^6^ cells/ml. Cells were added to an equal volume of PKH26 (4 µM) in Diluent C, and incubated for 5 min at room temperature. An equal volume of FCS was added following which the cells were resuspended in complete medium and washed four times. The stained cells were incubated in appropriate medium for 4 hours before using them in coculture experiments.

### Inhibition and blocking assays

Two hours prior to coculture with infected cells, immature or mature MDDC were pretreated with the inhibitor/blocker. Anti-DC-SIGN, and anti-DC-SIGN-R antibody were obtained from R&D Systems; Integrase inhibitor (118-D-24), T-20 Fusion inhibitor, TAK 779, AMD3100 were obtained through the NIH AIDS Research and Reference Reagent Program, Division of AIDS, NIAID, NIH; and all other inhibitors were obtained from Sigma-Aldrich.

### Immunofluorescence

At indicated time points, cells were adhered to glass slides, and fixed using 3.7% formaldehyde at room temperature for 10 min, and washed with PBS. Following three washes, slides were blocked with PBS containing 5% FBS. Surface staining was performed with primary antibody (DC-SIGN or CD28) (1∶100 dilution, R&D Systems, or BD Biosciences) for 1 hr at 4°C. Cells were washed 3 times with wash buffer, and incubated with rabbit anti-mouse IgG Rhodamine (RRX) (1∶400; Jackson ImmunoResearch, West Grove, PA) or donkey anti mouse Cy3 (1∶1000; Jackson ImmunoResearch, West Grove, PA) or donkey anti-rabbit Alexa 488 (1∶1000; Molecular Probes) for 1 h at room temperature followed by nuclear staining with Hoechst or DAPI. Confocal microscopy was performed using Olympus 1000 scanning confocal microscope from Olympus America at the Center for Biological Imaging, University of Pittsburgh. Final composites were constructed in Adobe Photoshop CS (Adobe, San Jose, CA).

### Assay for Integrated HIV-1 DNA

To evaluate the integrated DNA in infected DC, infected DC (based on EGFP+/DC-SIGN+ double positive) were sorted using the MoFlo sorter at UPCI Biocontainment Flow facility with the purity of 100%. Sorted DC were evaluated for integrated HIV-1 DNA by real time Alu-LTR PCR method as described in Butler et al [55]. Briefly Cellular DNA was extracted from sorted cells using Qiagen QIAamp DNA Blood Mini Kit, and 500 ng of DNA was used for PCR reaction, along with 50 nM forward primer, 300 nM reverse primer and 100 nM of probe. The sequence of primers and probes are described in Butler et al [55].

### Statistical analysis

The results were expressed as mean±standard deviation. The data were analyzed using the Student's t test for paired samples. Statistical evaluation of relation between antigen uptake and DC infection was performed using linear regression analysis and R^2^ value was calculated.

## Supporting Information

Figure S1 (A) Phenotypic and functional analysis of Immature and Mature DC. DC were differentiated from CD14+ monocytes as described in Materials and Methods, stimulated with 1µg/ml LPS, and stained with CD1a, CD80, CD83, CD86 and HLA-DR monoclonal antibodies or were incubated with FITC-dextran for 50 min at 37°C or 4°C, and analyzed by flow cytometry. Overlay of histogram shows surface expression of CD1a, CD80, CD83, CD86 and HLA-DR in Immature (blue) and mature DC (red). Isotype control is represented in black. (B) Histogram indicates FITC fluorescence in immature and mature DC at 37°C (green) or 4°C (blue), last panel shows overlay of histogram comparing FITC in immature (dashed blue) and mature (dashed red) DC at 37°C. (C) Schematic of HIV-1wt-EGFP and HIV-1 delta Env-EGFP proviral constructs denoting the position of EGFP and IRES. (D) Comparison of MFI of EGFP fluorescence in HIV-1wt-EGFP reporter virus infected and EGFP plasmid transfected Jurkat T cells.
(0.13 MB TIF)

Figure S2 DC infection vs antigen uptake. DC were cocultured with either HIV-1wt-EGFP reporter virus-infected Jurkat T cells or with Jurkat cells expressing EGFP protein. Post coculture, the cells were stained for DC-SIGN and analyzed by confocal microscopy. DC infection, represents DC cells productively infected and expressing EGFP (was measured by EGFP distribution throughout the cell); DC uptake, represents DC take up EGFP protein (exhibit the punctate pattern). Red, indicates DC-SIGN positive cells.
(0.94 MB TIF)
