# Supplementary figures and images for: HIV-1 Infection of DC: Evidence for the Acquisition of Virus Particles from Infected T Cells by Antigen Uptake Mechanism
Source: PLoS One. 2009 Oct 15;4(10):e7470. doi: 10.1371/journal.pone.0007470 (PMC2759578; doi:10.1371/journal.pone.0007470)

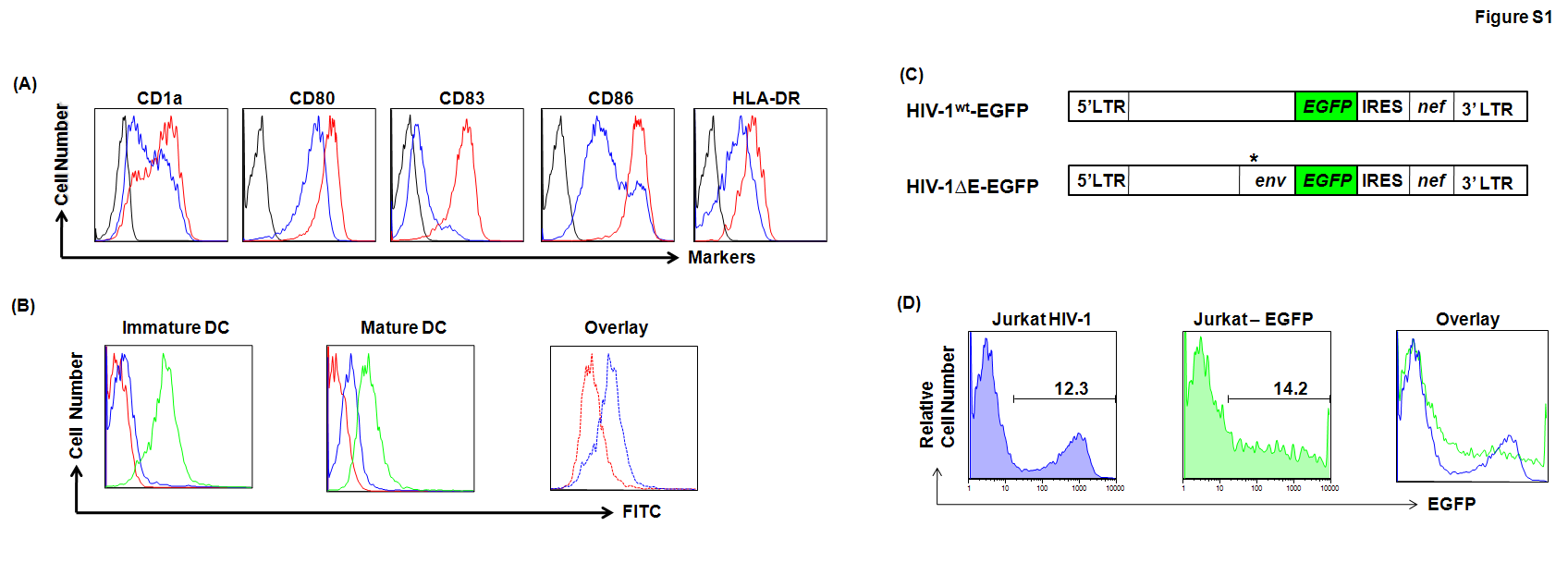

Supplement: Figure S1 — (A) Phenotypic and functional analysis of Immature and Mature DC. DC were differentiated from CD14+ monocytes as described in Materials and Methods, stimulated with 1µg/ml LPS, and stained with CD1a, CD80, CD83, CD86 and HLA-DR monoclonal antibodies or were incubated with FITC-dextran for 50 min at 37°C or 4°C, and analyzed by flow cytometry. Overlay of histogram shows surface expression of CD1a, CD80, CD83, CD86 and HLA-DR in Immature (blue) and mature DC (red). Isotype control is represented in black. (B) Histogram indicates FITC fluorescence in immature and mature DC at 37°C (green) or 4°C (blue), last panel shows overlay of histogram comparing FITC in immature (dashed blue) and mature (dashed red) DC at 37°C. (C) Schematic of HIV-1wt-EGFP and HIV-1 delta Env-EGFP proviral constructs denoting the position of EGFP and IRES. (D) Comparison of MFI of EGFP fluorescence in HIV-1wt-EGFP reporter virus infected and EGFP plasmid transfected Jurkat T cells. (0.13 MB TIF) [file pone.0007470.s001.tif]

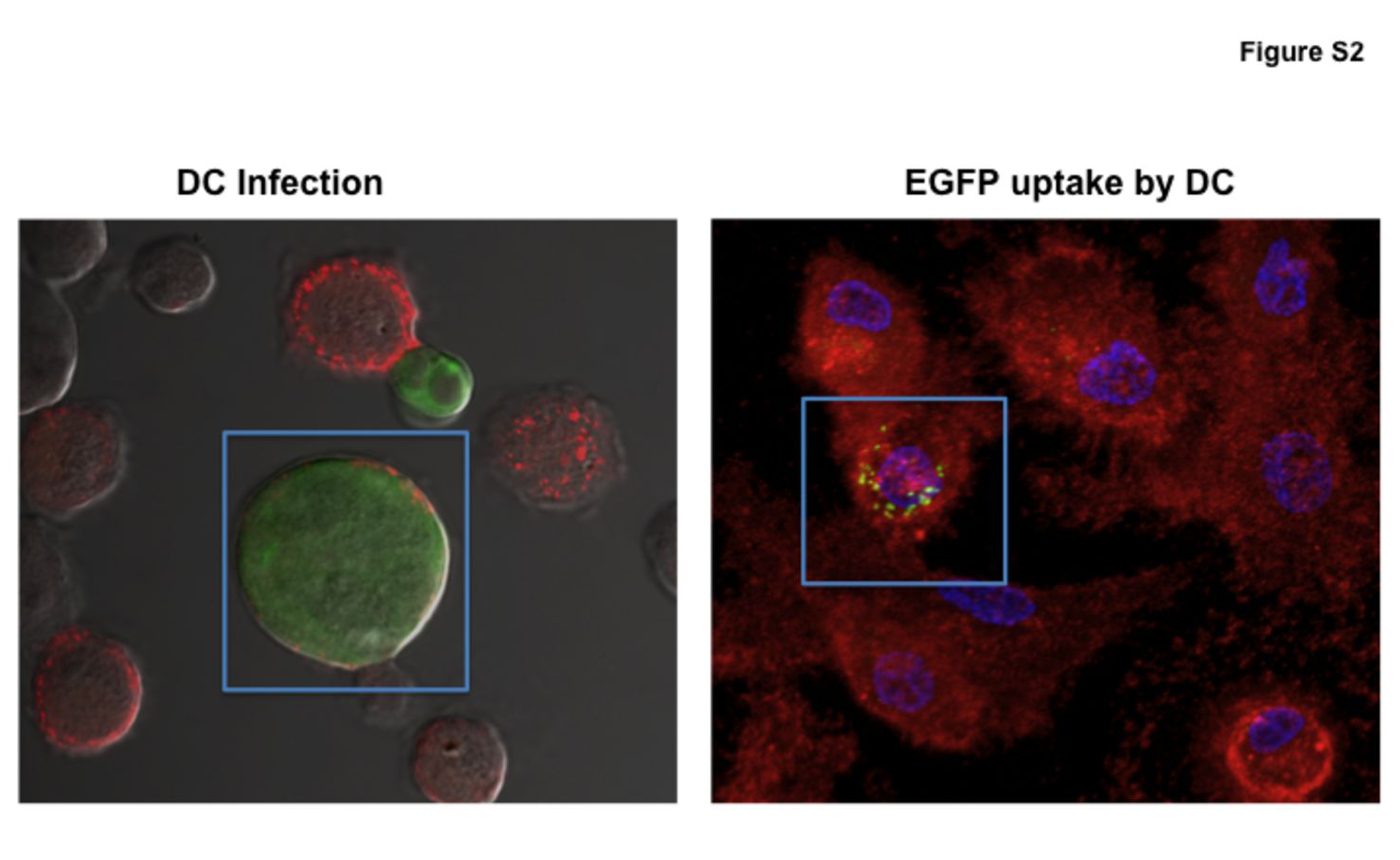

Supplement: Figure S2 — DC infection vs antigen uptake. DC were cocultured with either HIV-1wt-EGFP reporter virus-infected Jurkat T cells or with Jurkat cells expressing EGFP protein. Post coculture, the cells were stained for DC-SIGN and analyzed by confocal microscopy. DC infection, represents DC cells productively infected and expressing EGFP (was measured by EGFP distribution throughout the cell); DC uptake, represents DC take up EGFP protein (exhibit the punctate pattern). Red, indicates DC-SIGN positive cells. (0.94 MB TIF) [file pone.0007470.s002.tif]
